# “Just So You Know, It Has Been Hard”: Food Retailers’ Perspectives of Implementing a Food and Nutrition Policy in Public Healthcare Settings

**DOI:** 10.3390/nu13062053

**Published:** 2021-06-15

**Authors:** Kristy Karying Law, Claire Elizabeth Pulker, Janelle Diann Healy, Christina Mary Pollard

**Affiliations:** 1 East Metropolitan Health Service, Murray Street, Perth 6004, Australia; Kristy.law@health.wa.gov.au (K.K.L.); C.pollard@curtin.edu.au (C.M.P.); 2 School of Population Health, Curtin University, Kent Street, Perth 6102, Australia; Janelle.healy@curtin.edu.au

**Keywords:** public health nutrition, policy, food environments, food retail, food procurement, qualitative, implementation

## Abstract

Mandated policies to improve food environments in public settings are an important strategy for governments. Most Australian governments have mandated policies or voluntary standards for healthy food procurement in healthcare facilities, however, implementation and compliance are poor. A better understanding of the support required to successfully implement such policies is needed. This research explored food retailers’ experiences in implementing a mandated food and nutrition policy (the Policy) in healthcare settings to identify barriers, enablers, and impacts of compliance. Three 90-min workshops facilitated by two public health practitioners were undertaken with 12 food retailers responsible for operating 44 outlets across four hospitals in Perth, Western Australia. Workshop discussions were transcribed non-verbatim and inductive thematic content was analyzed. Three main themes were identified: (1) food retailers had come to accept their role in implementing the Policy; (2) the Policy made it difficult for food retailers to operate successfully, and; (3) food retailers needed help and support to implement the Policy. Findings indicate the cost of implementation is borne by food retailers. Communications campaigns, centralized databases of classified products, reporting frameworks, recognition of achievements, and dedicated technical expertise would support achieving policy compliance. Feasibility assessments prior to policy implementation are recommended for policy success.

## 1. Introduction

Globally, poor diet is a leading risk factor for non-communicable diseases including obesity, type 2 diabetes, cardiovascular disease, and some cancers. Few Australians eat a dietary pattern consistent with government dietary guidelines [1,2,3]. Two-thirds of Australian adults were categorized as overweight or obese in 2017–18, and only seven percent ate the recommended minimum amount of fruit or vegetables [4]. Food environments can influence what people eat, and hold the potential to reduce obesity and diet-related chronic diseases [5]. Food environments include ‘community’ environments (i.e., the number, type, and location of food outlets in a neighborhood); ‘consumer’ environments (i.e., what consumers encounter within a food outlet); and ‘organizational’ environments such as healthcare facilities which are the focus of this research [6].

Food procurement policies that aim to improve the availability of healthy food and drinks in organizational food environments such as hospitals are a promising public health initiative [7]. Governments can lead the implementation of healthy public food procurement policies in their owned healthcare facilities and hospitals [8]. Public hospitals are well placed to model and lead efforts to promote public health and prevent diet-related non-communicable diseases [9]. Policies should be evidence-informed, consider values, preferences, and resource implications, and be acceptable and feasible [8]. To be effective, policies should include nutrition criteria to identify healthy foods and drinks to encourage consumption, as well as foods and drinks to limit [8]. The World Health Organization recommends that nutrition criteria should be mandatory, specific, and enforceable [8]. Governments should support implementation through the provision of transition funds or incentives, supporting innovation, and providing ongoing technical expertise [10]. Implementation plans including communication, training, and provision of tools such as food catalogs or databases and monitoring checklists are also needed [8].

### 1.1. Healthy Food Procurement Policies for Australian Healthcare Facilities

In 2020, Australia’s Health Council provided nutrition criteria describing food and drink choices suitable for hospital and healthcare settings and called for action to implement them [11,12]. The standards apply to all food retail outlets including vending machines, ward trolleys, shops, cafes, and cafeterias, which are considered important sources of food for staff, visitors, and outpatients. Seven of the eight Australian state and territory governments have implemented standards for food and drink provision in healthcare facilities. Policies are mandated in the Australian Capital Territory [13,14], Northern Territory [15], South Australia [16], Western Australia (WA) [17], and Queensland [18]; and are voluntary in New South Wales (NSW) [19], and Victoria (VIC) [20].

Evaluation of Australian government healthy food procurement policies for healthcare facilities shows little impact to date. There was no change in the availability of healthier food and drinks in vending machines in NSW healthcare facilities in the Hunter New England region after implementing their ‘Healthier Choice’ mandated policy [21]. A later assessment of the implementation of the NSW ‘Healthy Food and Drink’ framework in two Sydney hospitals found no food outlets met all criteria for availability, placement, and promotion [22]. Queensland’s ‘A Better Choice’ mandated policy evaluation showed a quarter of the participating 134 public healthcare facilities had fully implemented the policy [23]. A 2021 review of Australian government hospital vending machine policies found poor compliance across all States [24].

Other Australian qualitative studies found high levels of support for such policies by parents in NSW [21], and; that strong working relationships between health promotion staff and stakeholders were important for policy implementation in a VIC hospital [25]. Given the lack of compliance to date, a better understanding of the support required to successfully implement these mandatory policies or voluntary guidelines for healthy food procurement is needed.

### 1.2. WA Healthy Food Procurement Policy for Healthcare Facilities

Since 2008, the WA Department of Health’s Healthy Options WA: Food and Nutrition Policy for WA Health Services and Facilities (the Policy) has been mandated in all healthcare facilities [26]. The Policy uses a traffic-light system, underpinned by nutrient criteria, to classify food and drink as green (healthy), amber (choose carefully), or red (unhealthy). All health services, facilities, and establishments owned or contracted by the WA Department of Health must meet three Policy criteria stipulating the proportions of green and red food and drinks permitted to be: (1) offered for sale, (2) displayed in food outlets, and (3) promoted (green food and drinks only). Criteria also apply to catering at meetings and events.

In May 2018, no WA hospital complied with the Policy. In response, the WA Health Minister instructed all health services’ food retail outlets to comply by 31 October 2018 and audited compliance in December 2018. The 2018–19 Statewide Audit report found no WA health service had achieved Policy compliance, although progress was made by individual food outlets [27]. A multi-choice questionnaire found concerns about loss of profits, difficulty in finding compliant products, and confusion about the Policy were barriers to implementation [27]. This information guided a Policy review, although there was no in-depth investigation of food retailers’ experiences in implementing the mandated policy. Following the review, the Policy was updated and released on 1 February 2021.

### 1.3. Implementation of WA Healthy Food Procurement Policy at East Metropolitan Health Service

The East Metropolitan Health Service (EMHS) in Perth, WA, is responsible for four public hospitals and one public-private partnership hospital which provides a combination of tertiary, secondary, specialist, and community health care services [28]. Baseline assessment of all food retail outlets at the largest EMHS hospital in July 2018 found only seven percent were compliant. Results were presented to the hospital’s food retail staff, volunteers, and the health promotion team, with tailored recommendations to achieve Policy compliance for each outlet. A quality improvement (QI) scheme was then developed to guide the approach taken to support policy implementation across the EMHS. The QI scheme ensures long-term management of policy compliance and ongoing improvement in food retail environments across the health service network and has been used by the Public Health Dietitian (PHD) to support policy implementation since 2018.

From July 2018, the PHD supported implementation across all the hospitals. Food retail staff, vendors, and volunteers were engaged through presentations describing compliance requirements and the importance of the Policy for public health. Monthly compliance audits and regular meetings with management, catering, and food service staff, volunteers, retailers, and vendors were conducted to assist with achieving and maintaining compliance. Food retailers provided information about potential new products or recipes for assessment against the Policy’s traffic light system prior to introducing them to sale. In the 2018–2019 Statewide Audit, 80% of all EMHS food outlets had achieved policy compliance [27]. By January 2020, 2600 individual products or recipes had been categorized and entered into a database. Internal audits determined that 91% of all EMHS food outlets were compliant with the Policy by August 2020. To achieve this, most food retailers had reduced the total number of snacks, removed unhealthy products, replaced some products with healthier options, and ceased the promotion of unhealthy food and drinks.

### 1.4. Impact of Implementing a Healthy Food Procurement Policy

Whilst healthy food procurement policies are important and relatively low-cost strategies for governments [29], little is published about the process of implementing them or their outcomes. Policy implementation is an emerging area of research and the impact of the change process on hospital food retailers is not known [30]. This study examines food retailers’ real-world experiences of implementing a mandated healthy food procurement policy across an Australian metropolitan health service. The experiences of catering staff, volunteers, retailers, and vendors (referred to as ‘food retailers’ hereon in) involved in implementing the mandated Healthy Options WA Policy across EMHS hospitals are examined. This study investigated food retailers’: (1) understanding of the Policy; (2) barriers and enablers to policy implementation; (3) impacts of the Policy, and (4) provides recommendations for ongoing policy compliance.

## 2. Materials and Methods

The exploratory nature of this research required a qualitative approach to encourage open-ended in-depth inquiry of the topic [31]. The workshop methodology is a useful way of exploring knowledge and experience [32]. Workshops allow participants to work together with researchers to discuss the current situation and generate ideas for implementing change [33]. The paradigm of social constructivism was applied when analyzing workshop data, to understand the experience of food retailers from their own perspective [32].

### 2.1. Setting

The setting for this study was four EMHS hospitals, in Perth WA. There were between two and 38 food outlets present in each of the four EMHS public hospitals, including one staff canteen, six cafes, 46 vending machines, two auxiliary shops, three ward trolleys, and three auxiliary kiosks.

### 2.2. Participant Recruitment

EMHS food retailers were invited to attend one of three 90-min workshops specific to their type of operation (i.e., health service operated, privately operated for profit, or volunteer operated for hospital fundraising). Food retailers from similar operational contexts were grouped together to encourage discussion and information sharing between participants.

Sixteen food retailers, who were responsible for operating a range of food outlets were identified from an existing contact list and invited to participate. Potential participants were contacted by email or phone and provided with a participant information sheet which included a brief description of the purpose of the research, and a consent form to sign and return prior to the workshop commencing.

### 2.3. Study Design

Three semi-structured small group workshops were conducted in November 2020 using meeting rooms available at the EMHS head office. A combination of presentations and group exercises was undertaken. KKL and CEP facilitated the workshops and research team members and two trained dietetic students were designated as note-takers for all three workshops.

On arrival, the workshop’s purpose was introduced and each group celebrated food retailers’ progress made towards achieving Policy compliance. Three presentations and three group exercises were conducted during the workshops, details are provided below.

The interactive presentations shared information, provided advice, and checked food retailers’ understanding of the Policy. The first presentation described the EMHS food system present across five metropolitan hospitals, with 71 food retailers, each with its own suppliers and governance structures. The presentation aimed to create a shared understanding of the setting in which the Policy was implemented. Participants were invited to comment on a map of the EMHS food system and make amendments based on any recent changes. An overview of the planned policy changes resulting from the state-wide review was then presented, including revised nutrition criteria and a new criterion for placement (i.e., prominence) of products. Each food retailer was provided with their product list showing the current traffic light rating (i.e., green, amber, red) and the new rating when the updated policy criteria were applied.

Two group exercises aimed to support participants to identify enablers of policy compliance, before moving on to identifying the barriers. In some workshops, the barriers were identified by participants when reflecting on enablers, and the flexible nature of the workshops allowed the facilitators to moderate accordingly. The first group exercise asked what had helped participants to implement the Policy, including any resources, tools, or people who had helped. The second group exercise asked participants how the Policy had impacted their operation, and what changes had been difficult. Participants were asked to describe specific examples of policy impact. After each exercise, they were invited to rank their top enabler and barrier to Policy compliance.

The final presentation, describing the process of analysis and recommendations, finished with an invitation to make comments and identify priorities for action.

Workshop discussions were transcribed non-verbatim by two researchers (CEP and JDH) and two dietetic students (MFFP and IF). Note-takers aimed to capture the meaning and approximate wording of participants’ remarks [34]. Field notes reported participants’ interactions, non-verbal communication cues, queries to follow up, and work produced by the participants. Data from each workshop were transcribed using Microsoft Word. Reflection and debriefing of the researchers (KKL, CEP, and JDH) immediately followed the conclusion of all three workshops, using triangulation to allow sense-making of participant responses [34]. Workshop transcripts were then de-identified before analysis.

The workshop plan is provided as a supplementary table (see Supplementary Materials Table S1).

### 2.4. Data Analysis

An inductive approach was used to analyze the data to identify the most important themes. Using a thematic coding approach, transcripts were manually coded by KKL and via NVivo by CEP. The process included familiarization with the transcripts, followed by identifying text segments relating to study objectives and labeling them with themes or categories. Coded text segments were cross-checked to reduce overlap among the themes or categories, with regular discussions and reviews of each researcher’s themes in an iterative way. Once there was general consistency in the themes, the final model incorporating the most important themes was created. The final list of important themes was discussed and agreed upon amongst all members of the research team.

## 3. Results

Twelve participants attended the three workshops. Three eligible participants declined due to other commitments or no response was given. Collectively, the workshop participants operated 44 outlets across four hospitals with all food outlet types represented.

Three main themes were identified: (1) food retailers had come to accept their role in implementing the Policy; (2) the Policy made it difficult for food retailers to operate successfully, and; (3) food retailers needed help and support to implement the Policy.

### 3.1. Food Retailers Had Come to Accept Their Role in Implementing the Policy

#### 3.1.1. It’s Fair When Everyone Has to Do It

Participants discussed the Policy from a position of acceptance, based on the effort they had already made to achieve compliance commencing in 2018. Fairness was integral to this viewpoint and discussed in all workshops. Participants said it was important that everyone was required to implement the policy at the same time. As one food retailer said:

“It’s better when everyone [i.e., all food retailers] does it at once, then it’s fair.”

(Participant 1)

Communication from the executive leadership was key to ensuring the implementation was seen to be fair and affected all food retailers equally:

“[The Chief Executive of EMHS] said ‘do it’ so we did. We thought the sky was going to fall in but it didn’t. It really helps when the person at the top says we have to do it, we have no choice.”

(Participant 5)

Food retailers also provided examples of practices they considered unfair because they disadvantaged their ability to be commercially competitive, inferring that the Policy did not seem to apply equally in all instances. They referred to food retailers located outside and near the hospitals that did not have to comply, stating staff and visitors would leave the hospital site to purchase the foods they could no longer sell. Occasionally food trucks were brought onto the site of one hospital, which they described as particularly unfair as they did not have to comply with the Policy. Staff and visitors were also able to bring unhealthy food or drink into the hospitals, as one participant pointed out:

“People can still bring in their own [soft drink] and other unhealthy foods on site and put it in their staff fridges.”

(Participant 6)

#### 3.1.2. Willing to Make Hard Changes

Participants were willing to make changes as part of their role in implementing the Policy. Some of these changes were noticeable to the customer such as changing the range of products sold, pricing, and display areas. Other changes to meet criteria were behind the scenes such as recipe modifications and vending machine stock-filling practices. Some changes were straightforward and in line with usual operations, whilst others were described as ‘hard’ and only made to help achieve compliance, as one food retailer explained:

“We introduced some new products to meet the Policy. It may not sell, and we know it won’t sell, but we need it to make the displays look good and meet the Policy…Just so you know, it has been hard.”

(Participant 8)

#### 3.1.3. Duplication of Effort Is Frustrating

Food retailers had accepted their role in policy implementation despite their frustration at the duplication of effort required to source and classify products against the Policy’s nutrient criteria. They said they understood that general product sourcing was part of their business, but the extra time spent looking for specific products to meet the Policy’s ‘green’ criteria was difficult. They were also frustrated because every food retailer had to repeat the process of identifying suitable products to source, which seemed a particularly inefficient use of time. Participants from all workshops said that an electronic list of available products that had already been rated against the Policy was needed. They said an electronic product list would make it easier for them to select suitable products and minimize duplication of effort:

“If everyone has to apply the system it’s not a good idea that everyone has to spend their time looking for products. Why can’t one person be responsible for sourcing the products and then we just pick what we want?”

(Participant 11)

### 3.2. The Policy Makes It Difficult for Retailers to Operate Successfully

#### 3.2.1. Pressures of Conflicting Demands

Participants spoke at length about the pressure of conflicting demands of the Policy, running a financially viable business, and meeting customer service demands. They referred to specific operational constraints they had to contend with such as supplier expectations, stock management, procurement logistics, and product wastage. With a raised voice, one retailer said:

“It’s financially unsustainable, but also environmentally unsustainable when the snacks go out of date and we have to waste them.”

(Participant 10)

Participants were notably frustrated when discussing the impact of the Policy on revenue. The need to make money was ranked as the top barrier to achieving policy compliance by participants. Whilst one retailer said sales were “busier” after making changes to comply with the Policy the majority were continually trying to balance policy requirements and commercial viability:

“We are not yet 100% compliant but I’m working on it. At the same time, I need to make money so if people don’t buy the products there’s no point in selling them.”

(Participant 2)

#### 3.2.2. At the Front Line with Customers

Food retailers gave examples of how they are at the front line with customers regarding the Policy. Participants openly shared examples of negative customer interactions they had experienced as a consequence of the Policy. Customer complaints were ranked as one of the top three barriers to implementing the Policy, with customer dissatisfaction with the lack of choice the main reason for the complaints. Participants felt that the Policy impacted the range of products available to the customer and described occasions when customers requested products that were no longer available or restricted due to the Policy. When recalling examples of customer abuse, there was strong agreement that the complaints were mostly from hospital staff. As described by one participant:

“We get a lot of abuse…Hospital staff are the worst because they know what we used to sell, visitors don’t know. Doctors, nurses, cleaners, any hospital staff can be aggressive with us and say, I work hard why can’t I have salami/3 meats in my sandwich/meal?”

(Participant 5)

To mitigate this, food retailers had trained their staff and volunteers to respond positively to customer queries or complaints. They gave examples of responses that used the Policy to explain the changes or they suggested alternative product options. Other responses attempted to shift customer expectations:

“I tell people we’re not a supermarket, we don’t have everything available.”

(Participant 3)

#### 3.2.3. Creates Extra Work for No Benefit

The additional burden of implementing a policy that required them to work in ways that are counter to their view of best retail practices was discussed. Participants gave examples of new stock filling practices and display infrastructure that had been introduced to support them to become compliant, going against usual practices. Staff constantly moved products around the food outlet space to meet the Policy ‘offer and display’ criteria ratios. Whilst food retailers had accepted this extra work was part of maintaining compliance, they were annoyed by the inefficiencies caused for little benefit:

“[The manager] organized infrastructure for us. There’s a fridge to display healthy snacks and meals but nothing sells. In the evening we clear it all out and put it in other display areas in other fridges. It just doesn’t sell from that one.”

(Participant 8)

“[in agreement] … It’s tricky, there’s a lot of mucking about with stuff to make sure we’re compliant even though it doesn’t sell. It creates work for no benefit.”

(Participant 5)

Participants also described the burden they experienced due to the expectation to carry out extra duties related to the Policy. They particularly expressed this when referring to sourcing and classifying products, and communications to staff and customers, which they considered to be beyond the scope of their role:

“That shouldn’t be our job to do that. We can’t go and say you can’t do this for example in the outpatients’ reception desk. It should come from a higher level. There are staff there who’ll say to us, who are you to tell us what to do?”

(Participant 3)

### 3.3. Food Retailers Needed Help and Support to Apply the Policy

#### 3.3.1. Confusion and Misinformation

The Policy requirements, especially the nutrient criteria that underpin the traffic light system, were confusing and participants said they needed help to understand how to achieve compliance. Both food retail staff and volunteers found it difficult to know which products were rated as ‘green’ or healthy according to the Policy’s criteria. Those who also worked across inpatient food services felt the differences between inpatient nutrition standards and the Policy added to the confusion. Food retailers also received misinformation from suppliers, as one participant commented:

“The last manager used to say suppliers would get pushy. They’d try to push products on her that weren’t suitable, so she had to educate the suppliers as well as the volunteers.”

(Participant 4)

#### 3.3.2. Timely Support from a Trusted Source Is Essential

Support from a trusted source was described as essential to help food retailers understand and implement the Policy. In all workshops, the PHD was ranked as the top enabler for compliance. Participants gave examples of how they relied on the PHD to help them classify products, analyze and modify recipes, suggest suitable products, and clarify misinformation. They also referred favorably to the snapshot reports of their food outlet’s product ratios which were provided by the PHD and used to inform the specific changes needed to achieve and maintain compliance. The value of this technical advice was enhanced by the timeliness of the PHD’s response. Inherent to the support provided was the relationship and trust that had been built between the PHD and food retailers since 2018. As one retailer explained:

“[KKL] is our number one help, we’ve sent everything through her…We get misinformation from the suppliers who tell us it meets the green nutrient criteria. We always check with [KKL] first.”

(Participant 5)

Other sources of support needed were to identify suitable products to sell, and sharing lists of ‘green’ products, paying external auditors for menu assessments or searching interstate automated databases for ‘green’ or healthy product suggestions.

#### 3.3.3. Sourcing Affordable, Acceptable ‘Green’ Products Is Hard

Food retailers wanted to offer healthier options but found it hard to source affordable and acceptable ‘green’ products. Those who had attempted to make their own products also faced difficulty meeting the nutrition criteria. Participants spoke at length about the lack of ‘green’ products available to them, particularly snacks, which were limited by their suppliers or food manufacturers:

“We don’t manufacture any snacks. If snacks are deleted by [major supermarkets] then they get deleted by the manufacturers…we have loads of amber products but only have nine green products.”

(Participant 10)

Affordability and profitability were equally as important when sourcing ‘green’ products. When they were able to find ‘green’ products, setting a price point was challenging. The price needed to be affordable for customers yet also profitable for the food retailer:

“[It’s] a challenge to find healthy things that people can afford to buy…there’s not much healthy [food] that you can buy in bulk that is long life…we started to make things, but staff contact went up and it became more expensive.”

(Participant 6)

Food retailers were frustrated by the difficulties they experienced in sourcing ‘green’ shelf-stable packaged products that would be both acceptable to the customer and suitable for the specific food retail outlet’s context as illustrated by the two participant comments below:

“We have the healthy pies that are reduced fat, the light range. People don’t like them and ask for the proper ones.”

(Participant 12)

“It’s been made too restrictive and no one buys the products. I don’t blame them, some of the stuff tastes like cardboard…At 3 am in the emergency [department], a tin of tuna is not going to cut it.”

(Participant 10)

#### 3.3.4. Some Rules Seem Wrong or Unachievable

Reactions to the updated Policy ranged from positive (nodding, open posture, leaning forward) to neutral (taking notes, concentrating without expression) to negative (arms crossed over chest, sighs). Participants were highly engaged during this presentation, and upon learning about the changes to the underpinning nutrient criteria, they immediately referred to their revised product categorization list to see how the new criteria had impacted their products’ traffic light rating. Participants described the changes to the Policy as concerning for two main reasons: firstly, they felt some of the policy criteria changes seemed wrong or inconsistent, and expressed disbelief at them:

“[questioning expression, flicking through papers] Some of the new product criteria seem strange, like making juice red but smoothies which include fruit are green…Hot chocolate will also be green, but fresh juice is red. It’s wrong.”

(Participant 1)

Secondly, participants were concerned about their ability to comply with the new mandatory requirements for how and where products could be placed within a food outlet. They questioned the process of quantifying an outlet’s display and why there were new product placement rules. They wanted real-life examples to help them understand how the new requirements would fit into the context of their own outlets and quickly gave examples where they felt compliance was not achievable. In response to a point-of-sale rule which states that only green items can be placed within arm’s reach of the cash register, one food retailer commented:

“That’s very difficult just because of space, our counter is only about 1.5m wide and that’s where all the products are displayed.”

(Participant 11)

## 4. Discussion

This study contributes Australian metropolitan health service food retailers’ real-world experiences of complying with a mandated healthy food procurement policy. Despite most Australian jurisdictions having a mandated policy or voluntary guidelines for food sold in healthcare facilities [35] compliance is very low, with a lack of resources and support, and difficulty sourcing healthy products and financial concerns cited [21,22,23]. This study presents food retailers’ perspectives on what is required to implement a mandated policy.

### 4.1. Barriers to Policy Implementation: Financial Viability and Customer Satisfaction

All food retailers described their two main priorities as running a financially viable business and having satisfied customers, which the Policy negatively impacted. Controlling costs of goods (i.e., food and drinks) and labor (i.e., staff) are critical to surviving in food retail and food service, which includes implementing procedures to ensure operations are efficient [36]. Delivering customer satisfaction is fundamental to food outlet operations, which is influenced by price, product choice, product quality, and appropriate customer service [37,38]. Whilst the Policy was designed to change retail practices to increase the availability and display of healthy food and drinks, an unexpected and disproportionate impact on food retailers’ business operations was described. Mandated government policy is considered a low-cost option [8,29] but the negative impact on food retailers’ workload, revenue, customer satisfaction, and usual commercial practices suggests that a cost is borne by those who the policy is regulating. Given the importance of these contextual factors, a cost-benefit analysis could inform the feasibility of policy implementation [29]. Addressing these factors during the policy development process is fundamental to successful policy implementation and compliance.

Food retailers discussed the challenges of responding to customer complaints or queries about the changes they had made to achieve policy compliance, including removing unhealthy products from sale. The frequency of having difficult conversations with customers had led to the development of specific training for some retail staff. An evaluation of implementing a mandatory standard for food products and promotions in Scottish hospitals identified the need for the policy owner to communicate with customers directly about the policy [39]. Food procurement policy should be accompanied with wrap-around initiatives to increase public awareness and support for changes. Initiatives such as communications campaigns can help to normalize the approach with customers and increase their acceptance of policy actions [29,40]. Increased public support for the provision of healthy food and drinks in healthcare facilities would likely assist with policy implementation and compliance.

### 4.2. Barriers to Policy Implementation: Lack of Understanding of the Policy

The complexity of the nutrient criteria underpinning the Policy was a key barrier to implementation and has been reported previously [35]. High levels of individual agency, cognitive resources, and time are required to understand and classify products against the 126-product group-specific criteria within the Policy [41]. Food retailers said they lacked the knowledge and skills to apply the nutrient criteria and technical support was essential. The specialist skills of dietitians or public health nutritionists are required to understand and interpret nutrient criteria as they apply to the procurement of packaged products and prepared meals [25]. To assist with policy implementation, governments should develop and update a centralized list or database of classified food products, which reduces the technical burden of compliance and assists food retailers in identifying healthy products [8,10]. For example, the VIC Healthy Eating Advisory Service database ‘FoodChecker’ includes thousands of products supplied from a very large, external database which are classified according to the Healthy Choices guidelines using an automated algorithm [42].

### 4.3. Enablers to Policy Implementation: Adopting a QI Approach

The findings of this study indicate that the reason why the food retailers overcame the challenges to achieve compliance was the QI approach taken by the EMHS to support policy implementation since 2018. The QI scheme was adapted from the Donabedian framework for healthcare quality assessment which uses a triad of criteria to evaluate quality: structure, process, and outcome [43]. Applied to this Policy, structure refers to the design, governance, and resourcing. Process denotes the activities undertaken to implement, monitor, and report on the Policy. Outcome measures include compliance and the impact on the food retail system; most commonly the availability, affordability, promotion, and purchase of food products. Structure influences process, which influences outcome [44]. The QI scheme also recognizes external factors that influence the health facility retail food system profile (e.g., food marketing and business practices, individual preferences) and their influence on dietary intake and nutritional health (see Supplementary Materials Figure S1).

Although the QI scheme was a behind-the-scenes framework, parts of the QI scheme developed to support policy implementation were identified by the food retailers when they described the importance of executive leadership to create a perception of fairness, a trusted technical expert, tools, and ongoing feedback with formal acknowledgment of progress. These results demonstrate how specific structure and process components of the QI scheme assisted food retailers to achieve policy compliance.

#### 4.3.1. Executive Support Led to a Perception of Fairness and Accountability

Fairness was identified as an important enabler of policy compliance. Addressing the value of fairness and what that means to food retailers appears to be critical to policy success. Executive-level support led to perceptions of fairness, as all food retailers were in it together. Since mandated policies can create a level playing field (i.e., apply to all hospital food retailers equally) this likely assists in policy acceptance [39]. A QI scheme also addresses gaps in management and accountability which have previously been identified as barriers to policy compliance [29,35]. Executive-level support and engagement was maintained through regular updates on policy compliance including barriers to progress. An individualized letter of Chief Executive endorsement was provided to compliant food retailers in recognition of their achievements. The recognition of achievements is a well-documented aspect of strong food policy accountability [45].

#### 4.3.2. The Dedicated Dietetic Resource and Trusted Expert

Adequate and committed resourcing was essential to support food retailers to meet policy requirements in the EMHS. The previous approach of periodic State-wide audits with a single compliance report and access to a website of resources did not result in Policy compliance. Inadequate resourcing has been identified as a hindrance to policy implementation and compliance, impeding public health impact and risking policy failure [29,35]. This research suggests that when food retailers are provided with dedicated support from a trusted technical expert, compliance is possible. Despite the challenges experienced by food retailers the motivation to comply remained high. This motivation is likely to be a result of the trust built between the dedicated personnel (PHD) and food retailers since 2018, supported by the QI approach. Health service staff who prioritize and invest time and effort to build rapport with food retailers have been found to be key to policy acceptance [25].

#### 4.3.3. Processes Were Established for Monitoring and Evaluation

The QI scheme identifies the process components needed to achieve and maintain compliance: regular monitoring and evaluation activities, corrective actions, reporting, and staff training. The food retailers recognized the need for regular nutrition and dietetics input for recipe analysis and product classification as well as extra staff training, and communications to improve understanding of the Policy. However additional activities were needed behind the scenes for successful policy implementation. Templates were used to provide tailored corrective actions for non-compliance and record monitoring activities and compliance progress. Health service-wide communications were implemented to recognize and share food retailers’ progress.

### 4.4. Importance of Assessing Feasibility of Implementing Mandated Policies

Mandated policies to improve food environments in public settings have been described as an important strategy for governments [8,10,29,40]. Voluntary policies and guidelines require the least government intervention [46] and supporters of the approach say they are lower cost, more flexible, and less adversarial than the alternative [47]. However, voluntary policies have been criticized as a way for food companies to avoid government mandates [48] which are required when there is a failure of the market to ensure customers purchase and consume recommended nutritious foods [49]. Legislation is considered a more effective way to implement change [50].

To date, there is little evidence that government policies to improve hospital food environments have been effective in Australia. This research demonstrates that food retailers require much more than a mandated policy to effectively bring about change. Clear governance structures and adequate resourcing to build system-wide capacity for implementation, monitoring, and enforcement mechanisms are essential. The World Health Organization’s Action Framework [8] recommends key steps for successful policy implementation, monitoring, enforcement, and evaluation. The recommendation by food retailers for a centralized database of classified products is consistent with this framework and would build capacity for policy implementation State-wide, likely avoiding duplication of effort and increased burden by other health services in the region.

This study also draws attention to the importance of assessing the feasibility of mandated policies prior to and during implementation. The barriers identified by the food retailers raise the question of how to resolve the conflicting priorities of business and health faced by food retailers located in healthcare facilities. The Policy appears to work on face value with respect to its aim of increasing the availability of healthier options. But there is a clear conflict between the fundamentals of the food retail business i.e., making a profit, commercial competition, and public health priorities i.e., obesity prevention [51]. This tension between conflicting demands, as well as new Policy requirements that appeared unachievable highlights the importance of developing policies that are fit for the context.

Future development of public procurement policies should address contextual considerations of food outlets within public settings and usual commercial operations. This is particularly relevant in Australia and other western countries, where hospital food retailers are not necessarily government employees but are small business owners, employees of for-profit organizations, or volunteers whose primary goal is fundraising. Policy development should also include feasibility testing to ensure real-world practicality and the best chance of implementation success [52]. Making changes to existing policies requires careful consideration, to manage food retailers’ progress and ongoing commitment. The challenges to implementing the updated Policy discussed by the EMHS food retailers raise genuine concerns about feasibility. Food retailers are an important stakeholder in policy feasibility testing during the consultation and/or implementation phases, which minimizes conflict of interest with policy goals [53]. A thorough and transparent evaluation of Policy outcomes is also recommended, to assist with justifying the ongoing need for change.

### 4.5. Strengths and Limitations

There are strengths and limitations to this study. The workshops brought together people with real-world experience of operating food outlets in a hospital setting who discussed the management, day-to-day operations, product procurement processes, and business decisions impacted by a healthy food procurement policy. The trust built with the PHD meant participants openly discussed their experiences, from a position of acceptance, not resistance, which contributes to an understanding of how food retailers can overcome challenges to policy implementation and achieve compliance. Almost all food retailers present had transformed their food outlets over a period of two years to become compliant with the mandated policy, a proportion the authors have not seen reported elsewhere in Australia. The modest sample size is a limitation of this study as it may not represent the views of the sector overall, however, a broad range of views were collected. The main themes identified were discussed in all workshops, despite differences in participant roles, operational procedures, and food outlet types. Although this qualitative research is limited to one Australian metropolitan health service, it highlights barriers and enablers faced by food retailers required to implement a mandated government policy that may be relevant to other health services. Findings are limited to the Australian policy, food retail, and healthcare system and may have limited application to international healthcare contexts. However, they can be generalized to other health services operating in WA that also need to comply with the Policy.

### 4.6. Implications for Healthy Food Procurement Policy and Practice

The findings from this study describe the challenges food retailers at healthcare facilities face in implementing and becoming compliant with a mandated healthy food procurement policy. Given the increasing focus of governments on implementing such policies in public settings, this research contributes an understanding of how to do this effectively in practice. As government policies become more complex, reflecting the increase in understanding about the impact of food environments on eating behavior, it is important to assess the feasibility of implementation to avoid policy failure. Feasibility assessments that determine whether the relevant government organizations have the capacity, resources, and authority required to undertake the extensive work required to develop, implement, and evaluate healthy food procurement policies are recommended [29]. In addition, external validity assessment of food provision that is compliant with mandated policies or voluntary guidelines would assist in building confidence in their ability to support healthy dietary patterns consistent with government guidelines. The impact of healthy food procurement policies on the food choice and dietary intake of target populations [54] and the economic impact of compliance on food retailers is also currently unknown.

## 5. Conclusions

Food retailers’ real-world experiences of complying with a mandated healthy food procurement policy across a metropolitan health service in Australia identified how they were supported to overcome challenges to normal business operations to achieve policy compliance, from a position of acceptance generated by strong policy leadership. The research demonstrated that when dedicated support from a trusted technical expert is provided as part of a QI approach, compliance is possible. Mandated policies must be feasible to be implemented, and although healthy food procurement policies are considered low-cost to governments, there is a cost of implementation borne by food retailers. Feasibility assessments should identify the resources, tools, structure, and mechanisms needed to support compliance and policy success. Specific tools and resources that would assist with policy implementation include communications campaigns to help normalize the approach and build customer acceptance; centralized provision of lists or databases of classified foods and drinks to reduce duplication of effort and the technical burden; reporting frameworks for monitoring, evaluation and feedback; and a systematic way of recognizing food retailers’ achievements.

## Supplementary Materials

The following are available online at https://www.mdpi.com/article/10.3390/nu13062053/s1, Table S1: Healthy Options WA Policy EMHS retailer workshop plan; Figure S1: A Health Facility Food Policy Quality Improvement System for Public Health.
