# Supplementary material for: “Just So You Know, It Has Been Hard”: Food Retailers’ Perspectives of Implementing a Food and Nutrition Policy in Public Healthcare Settings
Source: Nutrients. 2021 Jun 15;13(6):2053. doi: 10.3390/nu13062053 (PMC8232694; doi:10.3390/nu13062053)
Supplement: Supplementary file 1 [file nutrients-13-02053-s001.zip › Supp_Figure S1.pdf]

Figure S1: A Health Facility Food Policy Quality Improvement System for Public Health#

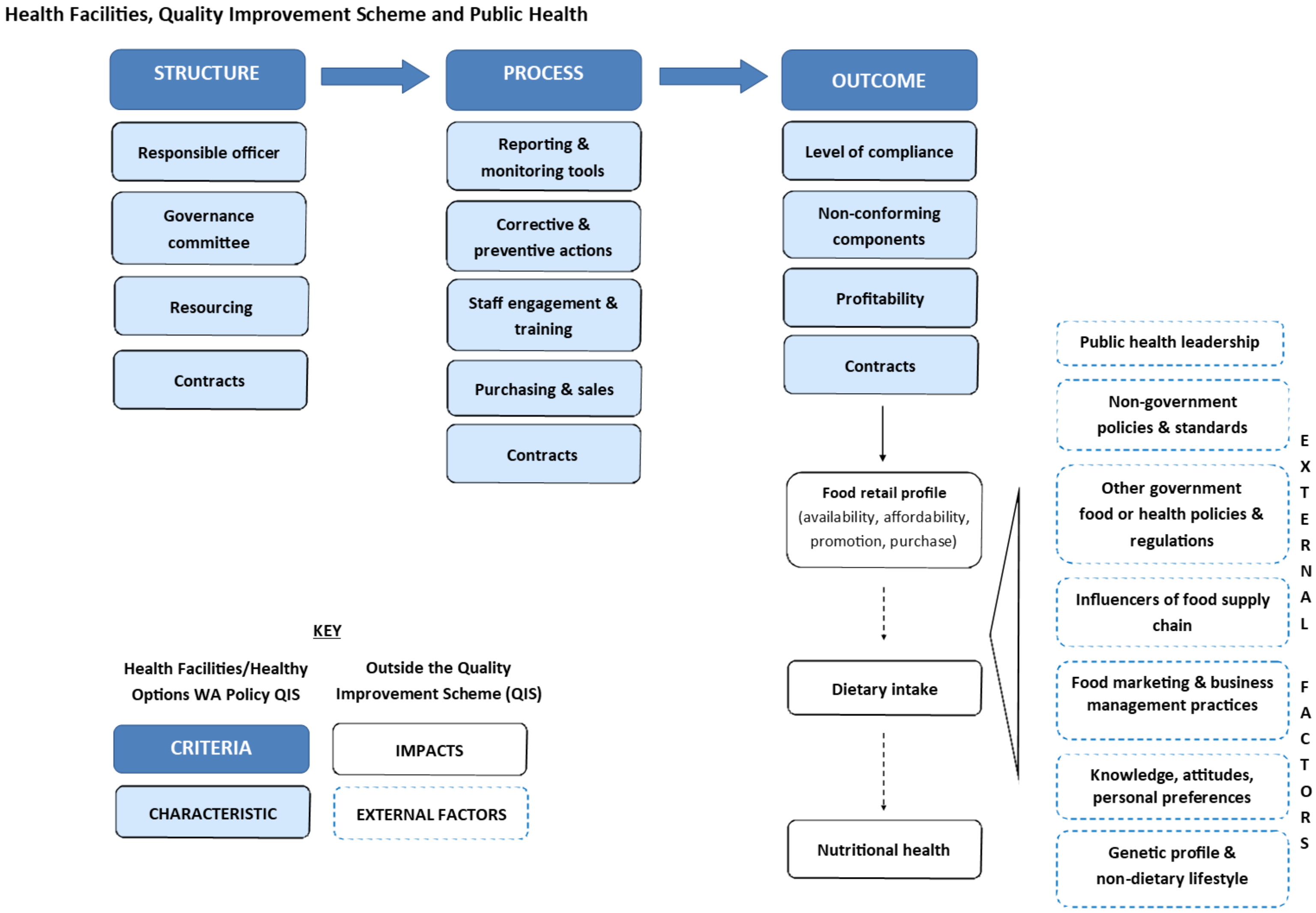

#Adapted from: Lawrence, M.A.; Pollard, C.M.; Weeramanthri, T.S. Positioning food standards programmes to protect public health: current performance, future opportunities and necessary reforms. *Public Health Nutr* 2019, *22*, 912-926, doi:10.1017/S1368980018003786.
