# Supplementary material for: “Just So You Know, It Has Been Hard”: Food Retailers’ Perspectives of Implementing a Food and Nutrition Policy in Public Healthcare Settings
Source: Nutrients. 2021 Jun 15;13(6):2053. doi: 10.3390/nu13062053 (PMC8232694; doi:10.3390/nu13062053)
Supplement: Supplementary file 1 [file nutrients-13-02053-s001.zip › Supp_Table S1.pdf]

Table S1: Healthy Options WA Policy EMHS retailer workshop plan

Content	Session Title	Session Content	Resources Required	Duration/Time
Introduction	Presentation 1a: Introduction to the workshop	Welcome and introductions<br>This presentation will describe the background to the workshop and what we aim to achieve, facilitate introductions between participants	*PowerPoint slides*	5 mins
Background	Presentation 1b:<br>Celebrating progress so far	Overview of EMHS' approach taken to support Policy implementation<br>Presentation of progress and success so far achieved by EMHS' food retailers, vendors, staff and volunteers	*PowerPoint slides*	10 mins
Obj2	Group exercise 1: What has helped food outlets to achieve Policy compliance? (identify enablers)	Participants will be asked to identify enablers:<br>• What has helped you to implement the Policy to achieve compliance?<br>• Key people (their roles and responsibilities) within your own operation and from EMHS that have helped you to achieve Policy compliance?<br>Wrap up: Participants will vote for their top enabler for compliance	*PowerPoint slides*<br>*Butchers paper*<br>*Markers*<br>*Coloured dots*	10 mins
Obj2, Obj3	Group exercise 2: What does the Policy require from food outlets? (challenges to compliance, impacts of Policy)	Participants will be asked to identify barriers and impacts:<br>• What have you needed to change about your operation to be compliant / what perceived changes have stopped you becoming compliant?<br>• Describe how this has impacted the way your food outlet is operated / how you perceive this would impact the operation?<br>Wrap up: Participants will vote for the most significant barrier to and impact of policy	*Post-it notes*<br>*Butchers paper*<br>*Markers*<br>*Coloured dots*	10 mins

Content	Session Title	Session Content	Resources Required	Duration/Time
Obj1	Presentation 2: EMHS food system mapping project	This presentation assists in setting the context for conversations that acknowledge that achieving compliance is complicated.<br>Describe the research project, present key findings.<br>Seek participants' input to comment, validate or amend the governance map as appropriate	*PowerPoint slides*<br>*A3 copies of governance map*<br>*Markers*	10 mins
Obj1	Presentation 3: Healthy Options WA Policy update	Presentation to inform retailers on the new 2020 Policy requirements, advice on new tools and resources, role of EMHS public health dietitian in providing technical expertise	*PowerPoint slides*<br>*Retailer product list*<br>*Food & Drink Guide*<br>*Placement resource*	20 mins
Obj4	Group exercise 3: What else is needed?	Group discussion to identify opportunities that will support retailers in achieving and maintaining Policy compliance, and any resources or technology required.<br>Wrap up: Participants to prioritise the most important actions	*Markers*<br>*Butchers paper*<br>*Coloured dots*	10 mins
Conclusion	Presentation 4: Next steps	Provide a brief overview of the analysis that will be conducted and actions EMHS will be taking.<br>Recommended next steps for retailers and available supporting resources/information	*PowerPoint slides*	5 mins
			**Total Time**	**80 mins**
